# Usability of serum hedgehog signalling proteins as biomarkers in canine mammary carcinomas

**DOI:** 10.1186/s12917-023-03761-7

**Published:** 2023-11-06

**Authors:** Haein Koo, Sungin Lee, Wan Hee Kim

**Affiliations:** 1 Department of Veterinary Clinical Sciences, College of Veterinary Medicine and Research Institute for Veterinary Science, Seoul National University, 1 Gwanak-Ro, Gwanak-Gu, Seoul, 08826 Republic of Korea; 2 Department of Veterinary Surgery, College of Veterinary Medicine, Chungbuk National University, Cheongju, Republic of Korea

**Keywords:** Hedgehog signal, Dog, Serum, Mammary gland tumour

## Abstract

**Background:**

The hedgehog signalling pathway has been implicated in tumourigenesis and progression of many tumour types. This pathway has recently emerged as a therapeutic target, and inhibitors of hedgehog signalling have gained considerable attention. In dogs, the roles of hedgehog signals in several types of tumours have been investigated, but their relationship with canine mammary gland tumours (MGTs) has not been established. This study aimed to evaluate the expression of sonic hedgehog (SHH) and glioma-associated oncogene 1 (GLI-1) in the serum and mammary tumour tissues of dogs.

**Results:**

SHH and GLI-1 protein expression levels were significantly higher in MGT tissues than in normal mammary gland tissues, as well as in malignant MGT specimens than in benign MGT specimens. Serum levels of SHH and GLI-1 were higher in MGT patients than in healthy controls (*p* < .001 and .001, respectively). Serum SHH level showed a statistically significant relationship with metastatic status (*p* = .01), and serum GLI-1 level showed a statistically significant relationship with histologic grade (*p* = 0.048) and metastatic status (*p* = 0.007). Serum hedgehog signalling protein levels were not significantly associated with breed size, sex, tumour size, or histologic type.

**Conclusions:**

Hedgehog signalling protein expression in canine MGT tissue and serum differed according to the histological classification (benign and malignant) and metastatic status, indicating a relationship between the hedgehog signalling pathway and canine MGT. Thus, the hedgehog signalling pathway may serve as a new biomarker and therapeutic target in canine MGT patients.

**Supplementary Information:**

The online version contains supplementary material available at 10.1186/s12917-023-03761-7.

## Introduction

Mammary gland tumours (MGTs) are the most common neoplasms in intact female dogs, with an incidence of up to 70%. Among patients with MGTs, the incidence of malignant tumours is 53.3%. Prognostic assessment is based on histologic grade, tumour diameter, and lymphatic or vascular invasion of distant metastases [1, 2]. The treatment of choice for MGTs is surgical resection, except in patients with inflammatory carcinoma or distant metastasis. Systemic treatment can be attempted in high-risk cases, but its efficacy has not been well established [1].

Hedgehog (Hh) signalling plays an important role in stem cell differentiation [3, 4] embryonic development [5], and tissue regeneration [6]. The Hh signalling pathway is activated by canonical and non-canonical mechanisms. The canonical mechanism is dependent on the primary cilium, where membrane receptor proteins and signalling components are concentrated. This pathway begins when the sonic hedgehog (SHH) ligand binds to the membrane receptor patched1 (PTCH1). In the absence of SHH, PTCH1 maintains its interaction with SMO (smoothened), which is inhibited by blocking its displacement to the cell membrane. Upon ligand binding, SMO enters the cell membrane and promotes the transcriptional activity of glioma-associated oncogene 1 (GLI-1). GLI-1 protein activation stimulates the expression of Hh target gene products such as PTCH1, GLI-1, Hh-interacting protein, and cell type-dependent genes such as cyclin D and E, Myx, and Sox2 [7–10]. In non-canonical Hh signalling, GLI-1 is activated by SMO-independent stimulation [11, 12].

The hedgehog pathway is required for normal development of the mammary glands, and is especially essential for the formation of the mammary buds [13]. The hedgehog ligands are expressed in the mammary epithelium, and PTCH1 is expressed in the epithelial and stromal components of the mouse mammary gland [10].

The Hh signalling pathway has been implicated in tumourigenesis and progression of many tumour types [14, 15]. In humans, activation of the Hh pathway has been linked to cancers of the brain, lung, prostate, pancreas, colon, and breast [7, 9, 16, 17]. The Hh signalling pathway has recently emerged as a therapeutic target, and inhibitors of Hh signalling, such as cyclopamine, sonidegib, and GANT-61, have gained attention [8, 9, 17].

In dogs, Hh signalling has been investigated for its roles in osteosarcoma and transitional cell carcinoma biology [18, 19]; however, the relationship between Hh signalling and canine MGT has not been established. Similarities between human breast cancer and canine MGT, including genetic, histologic, and clinical elements, have been identified [20]. The aim of this study was to evaluate the expression of SHH and GLI-1 in the serum and mammary tumour tissues of dogs. We hypothesised that Hh signalling elements would be more highly expressed in dogs with MGT than in normal dogs.

## Results

### Patients

A total of 68 dogs, including 25 healthy dogs and 43 dogs with MGTs, were enrolled in this study. The clinical parameters and histological findings of the dogs are presented in Table 1. The median age was lower in the control group (6 years; range: 2–11 years) than in dogs with benign (10 years; range: 7–17 years) and malignant (11 years; range: 8–13 years) MGTs. The major breed was the Maltese. Histological classification and grade were based on the criteria described by Goldschmit et al. [21] Two patients with oedema, erythema, firmness, and warmth of the mammary gland mass with distant metastasis were considered to have inflammatory carcinoma. Surgery was not performed in these patients, and histological diagnosis was not possible. Regional or distant metastases were confirmed in six patients with malignant MGT.

**Table 1:** Comparison of clinical parameters between healthy dogs (controls) and dogs with benign and malignant MGTs

	**Control (*n* = 25)**	**Benign tumour (*n* = 26)**	**Malignant tumour (*n* = 17)**
Age, years	6 (6)	10 (2.75)	11 (2)
Sex (n)	Female (8) Spayed female (17)	Female (11) Spayed female (15)	Female (9) Spayed female (8)
Breed (n)	Maltese (7) Pomeranian (4) Golden retriever (3) Bichon frise (2) Mixed (2) Poodle (2) Chihuahua (1) Samoyed (1) Shih-tzu (1) Pekingese (1) Yorkshire terrier (1)	Maltese (12) Poodle (4) Mixed (2) Shih-tzu (2) Pomeranian (2) Bichon frise (1) Cocker spaniel (1) Jindo dog (1) Samoyed (1)	Maltese (4) Shih-tzu (3) Poodle (3) Beagle (1) Chihuahua (1) Cocker spaniel (1) Mixed (1) Pomeranian (1) Schnauzer (1) Spitz (1)
Body weight (kg)	3.44 (2.13)	3.875 (3.68)	4.60 (3.62)
Histologic classification (n)		Adenoma, simple (9) Adenoma, complex (11) Benign mixed tumour (6)	Carcinoma, simple (10) Carcinoma, complex (1) Carcinoma, mixed (3) Carcinoma, solid (1) Inflammatory carcinoma (2)
Histologic grade (n)			Grade 1 (12) Grade 2 (3) Grade 3 (0)
Metastasis (n)			6

Continuous variables are presented as median and interquartile range

*MGT* mammary gland tumour

### Western blot

**Fig. 1:**
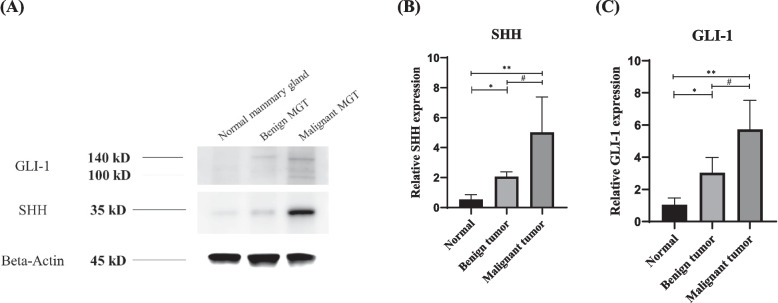
Comparison of SHH and GLI-1 expression in the normal mammary gland and MGT tissues. **A** For western blotting, the blots were cut prior to hybridisation with antibodies. SHH (35 kDa) and GLI-1 (100–140 kDa) protein levels in the normal mammary gland and benign and malignant mammary gland tissues. Beta-actin was used as the loading control. **B** Relative quantitation of SHH and (**C**) GLI-1 proteins were normalized to that of the control group. The mean and standard error of the mean are used to express quantitative data. (*, **, #: *p* < .05). SHH, sonic hedgehog; GLI-1, glioma-associated oncogene 1; MGT, mammary gland tumour

Western blotting was performed to confirm the expression of Hh signalling proteins in normal mammary glands and MGT tissues (Fig. 1A). Relative quantitation was calculated, and comparison was based on the normal sample. SHH (Fig. 1B) and GLI-1 (Fig. 1C) expression levels were greater in MGT tissues than in normal tissues and were significantly higher in malignant MGTs than in benign MGTs (*p* < 0.05).

### Serum levels of SHH and GLI-1 in healthy dogs and dogs with MGT

The median serum SHH and GLI-1 levels in the 25 healthy dogs were 2.38 ng/mL [95% confidence interval (CI), 1.23–3.53 ng/mL] and 17.03 ng/mL (95% CI, 10.65–23.41 ng/mL), respectively, and were significantly lower than those in the MGT group (4.53 ng/mL; 95% CI, 3.29–5.77 ng/mL and 28.19 ng/mL; 95% CI, 21.09–35.30 ng/mL, respectively; *p* < 0.001 and < 0.001, respectively).

**Fig. 2:**
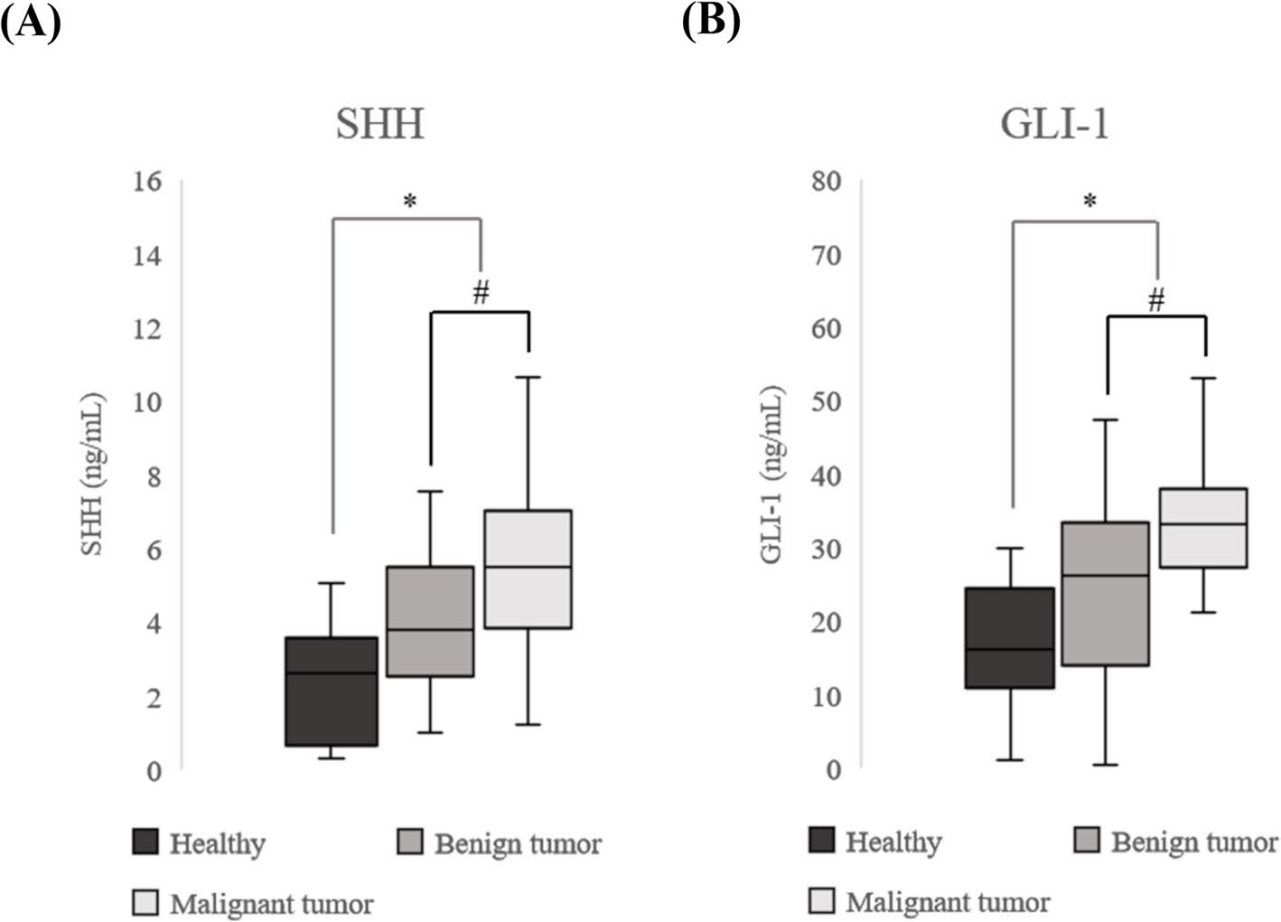
Serum SHH and GLI-1 levels in healthy dogs and dogs with MGT. **A** The serum SHH level was higher in dogs with MGT than in healthy dogs. **B** The serum GLI-1 level was higher in dogs with MGT than in healthy dogs. (*, #: *p* < .05). SHH, sonic hedgehog; GLI-1, glioma-associated oncogene 1; MGT, mammary gland tumour

To evaluate the association between Hh expression and disease severity, patients with MGT were divided into benign and malignant tumour groups (Fig. 2A and B). The median serum SHH and GLI-1 levels of 26 dogs with benign MGTs were 3.96 ng/mL (95% CI, 2.60–5.32 ng/mL) and 24.26 ng/mL (95% CI, 15.21–33.31 ng/mL), respectively. In 17 dogs with malignant MGTs, the median serum SHH and GLI-1 levels were 5.40 ng/mL (95% CI, 3.60–7.20 ng/mL) and 34.21 ng/mL (95% CI, 25.14–43.29 ng/mL), respectively. The expression levels of SHH and GLI-1 were significantly higher in the malignant MGT group than in the benign MGT group (*p* = 0.033 and 0.006, respectively).

### Relationship between Hh signalling protein expression and clinicopathological factors

The relationship between Hh signalling protein expression and clinicopathological features is shown in Table 2. Among dogs with malignant tumours, SHH and GLI-1 expression was significantly higher in metastatic patients (*p* = 0.01 and 0.007, respectively). Dogs with regional or distant metastases were included in the metastatic group. The serum level of GLI-1 expression was correlated with histological grade (*p* = 0.048), but the median SHH level was not (*p* = 0.093). The protein expression levels showed no correlation with clinicopathological parameters such as tumour size, sex, breed size, or histological type.

**Table 2:** Comparison of hedgehog signal expression associated with clinicopathological parameters

	**Number of patients (n)**	**SHH (ng/mL)**	***P* value**	**GLI-1 (ng/mL)**	***P* value**
Sex					
Female	20	4.17 ± 2.42		29.31 ± 10.11	
Spayed female	23	4.84 ± 1.71	.165	27.22 ± 13.40	.903
Breed size					
≤ 10 kg	36	4.56 ± 2.10		27.50 ± 12.47	
> 10 kg	7	4.39 ± 2.08	.936	31.78 ± 8.06	.468
Tumour size					
≤ 3 cm	24	4.03 ± 2.01		27.28 ± 9.48	
3–5 cm	7	4.88 ± 2.83		27.43 ± 16.56	
> 5 cm	12	5.32 ± 1.54	.154	30.47 ± 13.95	.534
Benign MGT					
Simple	9	4.69 ± 1.72		26.21 ± 12.23	
Complex	11	3.21 ± 1.47		22.79 ± 12.76	
Mixed	6	4.25 ± 1.91	.150	24.02 ± 10.76	.925
Malignant MGT					
Simple	10	4.32 ± 1.66		31.29 ± 7.02	
Complex	1	± 7.67 ±		± 32.92 ±	
Mixed	3	5.41 ± 2.23	.175	29.51 ± 5.36	.925
Histologic grade					
Grade 1	12	4.72 ± 2.01		30.00 ± 5.58	
Grade 2	3	5.85 ± 0.28	.448	37.94 ± 65.54	.048*
Metastasis					
Metastatic	6	7.26 ± 1.79		42.96 ± 9.81	
Non-metastatic	11	4.39 ± 1.91	.01*	29.44 ± 5.16	.007*

Continuous variables are presented as mean and SD

^*^P value < .05 indicates that the clinicopathological variable is considered to be statistically significant

### Survival curve

**Fig. 3:**
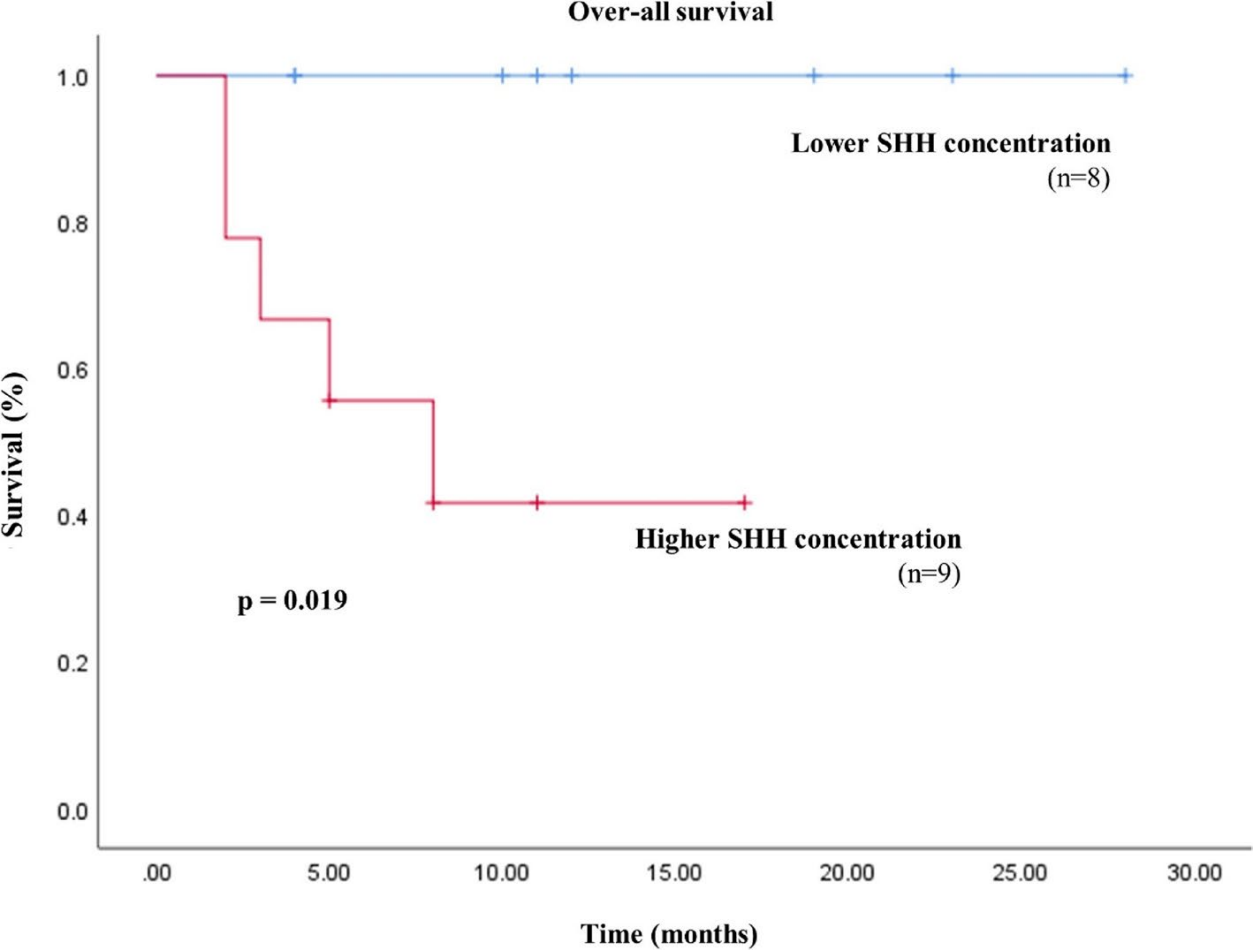
Kaplan–Meier survival curve of overall survival based on the sonic hedgehog (SHH) levels in 17 dogs with a malignant mammary gland tumour

Survival curves of 17 dogs with malignant mammary gland tumours were analysed using a Kaplan–Meier analysis (Fig. 3). Five dogs died during the study period, and all dogs died due to mammary gland tumours. The remaining 12 dogs were alive during the study. These dogs were divided into two groups: 8 dogs with higher SHH levels, and 7 dogs with lower SHH levels. The analysis of the survival curves showed a statistically significant association between high levels of SHH and poor overall survival (*p* = 0.019).

## Discussion

Dysregulation of the Hh signalling pathway has been proven to be involved in the initiation and progression of various types of tumours [14, 15, 22, 23]. Hh pathway inhibitors have been clinically developed for the treatment of several tumour types [24]. Recent studies have investigated the overexpression of Hh signals in human breast cancer cell lines [25]. In canines, several studies have been performed to determine the expression of Hh pathway mediators in different types of tumours [18, 19, 26]. The results of the current study suggest a relationship between Hh signalling pathway and canine MGTs.

Since SHH and GLI-1 are known to be prognostic markers in human breast cancer [27, 28] and GLI-1 is a critical transcriptional factor, we evaluated the expression of SHH and GLI-1 in normal dogs and dogs with MGT to investigate the relationship between the Hh signalling pathway and canine MGTs. Western blotting of the canine normal mammary gland and MGT tissues was performed to establish the likely sources of SHH and GLI-1 production and secretion in the serum. Previous studies have indicated that both epithelial cells and stromal fibroblasts produce Hh signalling proteins [22, 23]. The expression of SHH and GLI-1 in mouse mammary glands and human breast has also been described previously. In addition, their expression was reported to be higher in breast cancer tissue than in normal tissue [25, 29–31]. Our results showed that Hh expression was significantly higher in canine MGT than in normal mammary gland tissue. Malignant MGT showed higher Hh signal protein expression than benign MGT. These results indicate the potential relationship between the Hh signalling pathway and canine MGT.

On the basis of the western blot results, we assumed that the levels of serum SHH and GLI-1 would be higher in dogs with MGT than in normal dogs. The expression was significantly higher in dogs with malignant MGT than in those with benign MGT, as well as in dogs with MGT than in normal dogs. Increased SHH and GLI-1 protein levels in the serum of patients with MGT suggest that these factors may serve as biomarkers.

Correlation analyses were performed between the expression of Hh signal proteins (SHH and GLI-1) and clinicopathological parameters, which are known risk or prognostic factors [1, 32]. Patients with inflammatory carcinoma have significantly higher SHH and GLI-1 levels than those with non-inflammatory MGT because patients with inflammatory carcinoma have signs of systemic illness and distant metastases [1]. In our study, serum GLI-1 was positively correlated with histological grade; however, the correlation between SHH and histological grade was not statistically significant. In general, canonical activation of the Hh signalling pathway, which is initiated by Hh ligand binding, occurs mainly in cancers and cancer stem cells. In the non-canonical Hh signalling pathway, GLI-1 activity is induced independently of the presence of the Hh ligand [12]. It has been reported that several mechanisms of non-canonical activation may co-exist [9, 11, 24]. There is a lack of information on the role of non-canonical Hh signalling in canine MGT, and the results of serum GLI-1 and SHH levels based on the histologic grade suggest a relationship between the non-canonical pathway of Hh signalling and canine MGT. Further studies are required to confirm this hypothesis. Serum SHH showed a positive correlation with metastatic status. These data are consistent with previous reports in humans [27]. Serum GLI-1 levels and metastatic status were also statistically correlated.

Our findings demonstrated that higher serum SHH concentration patients showed poor overall survival than lower serum SHH concentration patients (*p* = 0.019). In human breast cancer, high serum SHH levels were associated with poor overall survival [27]. The result of this study imply the possibility of SHH as a valuable prognostic marker in canine mammary gland tumours.

This study has several limitations. First, the serum sample sizes of normal dogs and dogs with MGT were relatively small. The correlations based on tumour grade and molecular classification were limited. A larger sample size would help to obtain more reliable results and a multi-perspective analysis. Second, histopathological examination is limited to normal mammary tissue samples. To that end, healthy mammary glands were chosen from the total mastectomy specimens. To ensure that the tissue was as normal as possible, we chose glands that were at least one gland away from the mammary glands in which the mass was palpated. Third, the presence of Hh signalling proteins (SHH and GLI-1) in normal mammary glands and MGTs was confirmed only at the protein level. Further investigations, such as reverse transcription–polymerase chain reaction to identify its presence at the mRNA level and immunohistochemistry to confirm protein location, are needed to address these issues. Also, oestrogen expression has been shown to be correlated with Gli-1 expression, and the oestrogen receptor regulates non-canonical hedgehog signalling. Immunohistochemistry of mammary gland tissue for the oestrogen receptor and evaluation of the patient’s serum hormonal levels would advance our understanding of the Hh signalling pathway in patients with MGT. In addition, only a portion of the Hh signalling proteins (SHH and GLI-1) was observed. Examining the expression of other Hh signalling pathway factors would improve therapeutic options for other Hh pathway inhibitors.

In conclusion, our results suggest that the Hh signalling pathway is associated with canine MGT. We observed that Hh signal protein expression in the serum was higher according to the histologic classification (benign or malignant) and metastatic status. Our study opens up the possibility of using the Hh signalling pathway as a new biomarker and therapeutic target in canine MGT patients. Further studies are needed to understand the specific mechanism by which the Hh signalling pathway promotes the development of canine MGTs.

## Materials and methods

### Patient selection

A total of 68 female or spayed female dogs were selected for this study (age: 2–17 years), which included 43 patients with MGTs and 25 healthy controls. All dogs were recruited from the Veterinary Medicine Teaching Hospital at Seoul National University (VMTH-SNU) between April 2020 and April 2022. Physical examination, complete blood count, serum biochemistry, and abdominal ultrasonography were performed to confirm that all healthy controls were normal. Patients with MGT were assessed through physical examination. Three-view thoracic radiography and abdominal ultrasonography were performed to detect their metastatic status. Complete blood count and serum biochemistry were performed before surgery. Patients with MGT underwent surgery at VMTH-SNU. They were diagnosed based on the findings of histopathological examinations conducted at IDEXX Laboratories. CT scans were performed in patients diagnosed with a malignant mammary gland tumour. Two patients with an oedema or a firm and warm mammary gland mass underwent fine-needle aspiration; subsequently, mammary adenocarcinoma with necrosis and inflammation were found. In addition, the results of the fine-needle aspiration of the medial iliac lymph node showed cells with the same cell morphology as the cells in the mammary gland mass. These patients were diagnosed with inflammatory carcinoma, and surgical resection was not conducted. The patients’ clinical data, tumour size, histopathological type, and metastasis were all collected. In addition, follow-up data regarding survival time in the malignant MGT group was obtained telephonically in August 2022. Overall survival was calculated from the time of primary surgical treatment to that of death due to the MGT.

### Collection of blood samples and tissue specimens

Blood samples were collected from the jugular vein, centrifuged at 1000 xg for 15 min to separate the serum, and stored at -80 °C; the collected blood samples were used to measure GLI-1 and SHH levels. Normal mammary gland and benign and malignant MGT tissue specimens were collected from dogs that underwent surgery between April 2020 and November 2021 at VMTH-SNU.

Cefazolin (22 mg/kg, IV) was given 30 min prior to surgery, and the patients were premedicated with midazolam (0.2–0.4 mg/kg, IV). General anaesthesia was induced with propofol (6 mg/kg, IV) and maintained with isoflurane gas. Ketamine-remifentanil constant rate infusion was administered during surgery to control pain.

Mammary tissue samples were cut into a volume of 5 mm^3^, and then were promptly frozen in liquid nitrogen and stored at -80 °C until used for western blotting. After suturing the resected area, mastectomy tissues were fixed for 24–48 h in 10% neutral buffered formalin, and then sent to IDEXX Laboratories for analysis. A histopathological examination was performed for all palpated masses.

### Measurement of serum GLI-1 and SHH

Serum levels of GLI-1 and SHH were measured in duplicate using canine GLI-1 (Cat no: MBS7200887) and SHH (Cat no: MBS738808) ELISA kits (MyBioSource) according to the manufacturer’s protocols. Briefly, 100 μL of serum and/or standards were added to the coated wells. Further, 50 μL of conjugate was added and mixed well. The plate was incubated for 1 h at 37 °C. The solution was then discarded, and the wells were washed with 1 × wash solution five times. Further, 50 μL of substrate A and 50 μL of substrate B were added and incubated for 20 min at 37 °C. Subsequently, 50 μL of stop solution was added, and the absorbance of each well was measured at 450 nm using a microplate reader.

### Western blotting

Proteins were extracted from the normal canine mammary gland and MGT tissue using RIPA buffer (Merck Millipore) with protease inhibitors (Sigma-Aldrich). Proteins were quantified using a BCA Protein Assay Kit (Bio-Rad), and absorbance was measured using a microplate reader at 570 nm. For denaturation, the extracted proteins (23 μg) were mixed with sodium dodecyl sulphate (SDS) loading buffer (GenDEPOT) and boiled at 100 °C for 5 min before loading. Equal amounts of protein were separated using 10% SDS polyacrylamide gel electrophoresis and transferred to a polyvinylidene difluoride membrane. The blots were cut prior to hybridisation with antibodies. The following antibodies were used in the western blotting assay: rabbit polyclonal primary antibodies against GLI-1 (1:1000, Novus Biologicals; Cat no: NB600-600) and rabbit polyclonal primary antibodies against SHH (1:500, LSBIO; Cat no: LS-C40460). The secondary antibody used was anti-rabbit IgG (1:5,000, GenDEPOT; Cat no: SA002-500). Protein expression was detected by chemiluminescence using an enhanced chemiluminescence detection reagent and visualised using an ImageQuant LAS 4000 mini biomolecular imager (GE Healthcare Bio-Sciences) (Additional file 1).

### Statistical analysis

All statistical analyses were performed using SPSS software version 25.0. The relative protein expression of SHH and GLI-1 determined by western blotting were analysed using the Kruskal–Wallis test. When significant differences were observed, the Mann–Whitney U-test was performed to compare each group. Normality tests were performed using the Shapiro–Wilk test. The Tukey–Kramer method was used to compare serum levels of SHH and GLI-1 among the three groups (healthy, benign MGT, and malignant MGT). Correlations of SHH and GLI-1 levels with clinicopathological parameters (sex, breed size, metastatic status, and histologic grade) were analysed using the Mann–Whitney U-test. The Kruskal–Wallis test was used to compare serum SHH and GLI-1 expression among histological types and tumour sizes. Kaplan–Meier survival curves were plotted and compared using the log-rank test. All data were expressed as mean ± standard deviation. *P*-values < 0.05 were considered statistically significant.

### Supplementary Information

**Additional file 1.**

**Additional file 2.**

## Data Availability

The datasets used and/or analysed during the current study are available from the corresponding author on reasonable request.
