# Supplementary material for: Usability of serum hedgehog signalling proteins as biomarkers in canine mammary carcinomas
Source: BMC Vet Res. 2023 Nov 6;19:231. doi: 10.1186/s12917-023-03761-7 (PMC10626804; doi:10.1186/s12917-023-03761-7)
Supplement: Supplementary file 1 — Additional file 1. [file 12917_2023_3761_MOESM1_ESM.pdf]

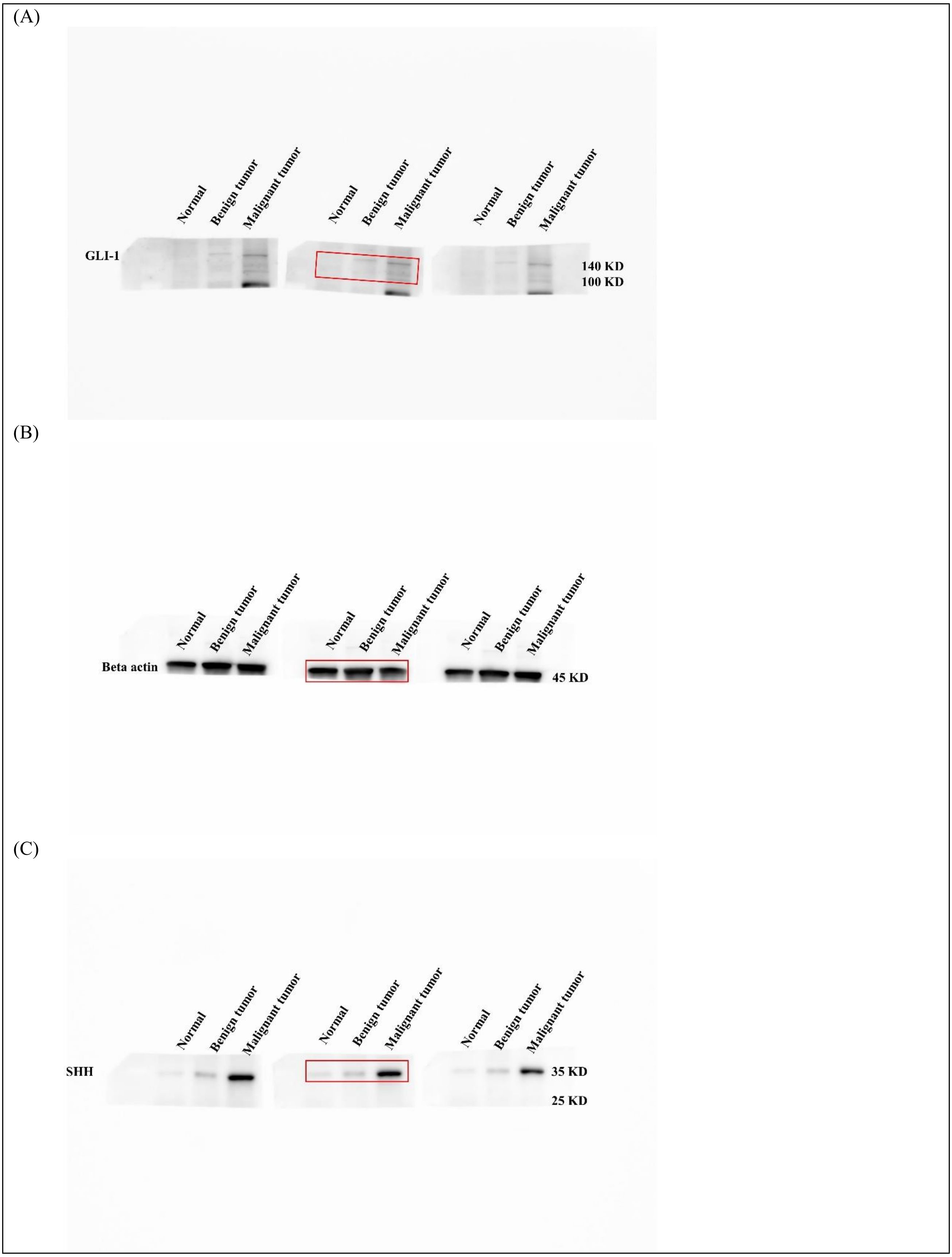

Supplemental Figure 1. Full length blots of GLI-1(A), beta actin1(B), and SHH (C) westen blotting. Red boxes indicate the cropped blots shown in Figure 1.

(A)

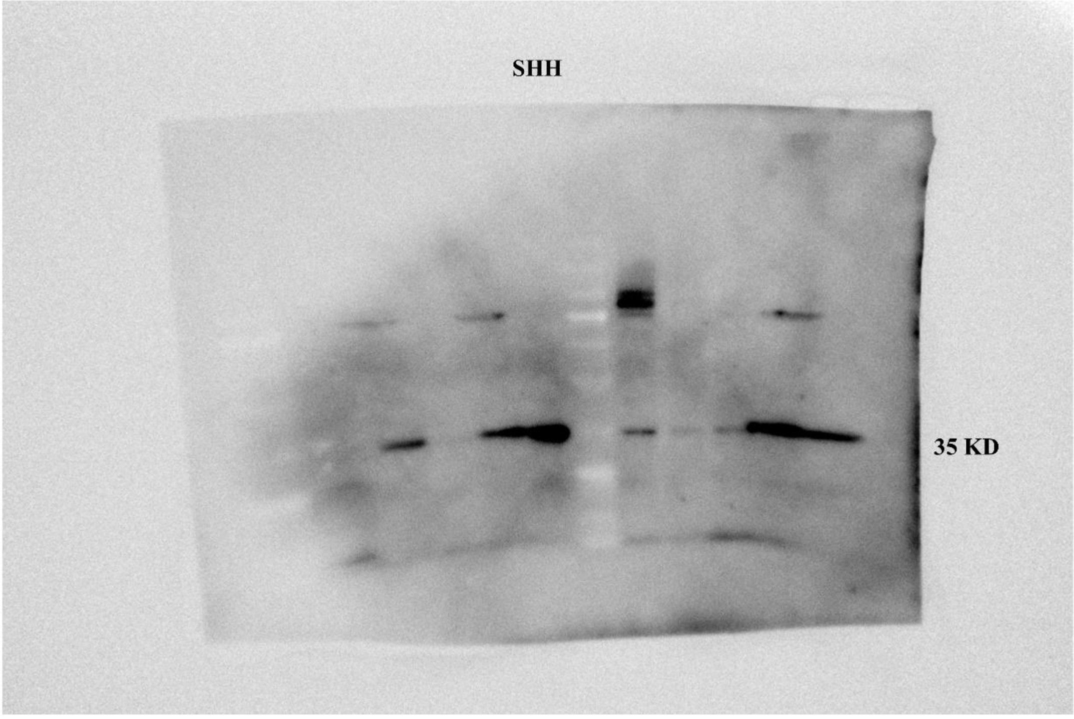

(B)

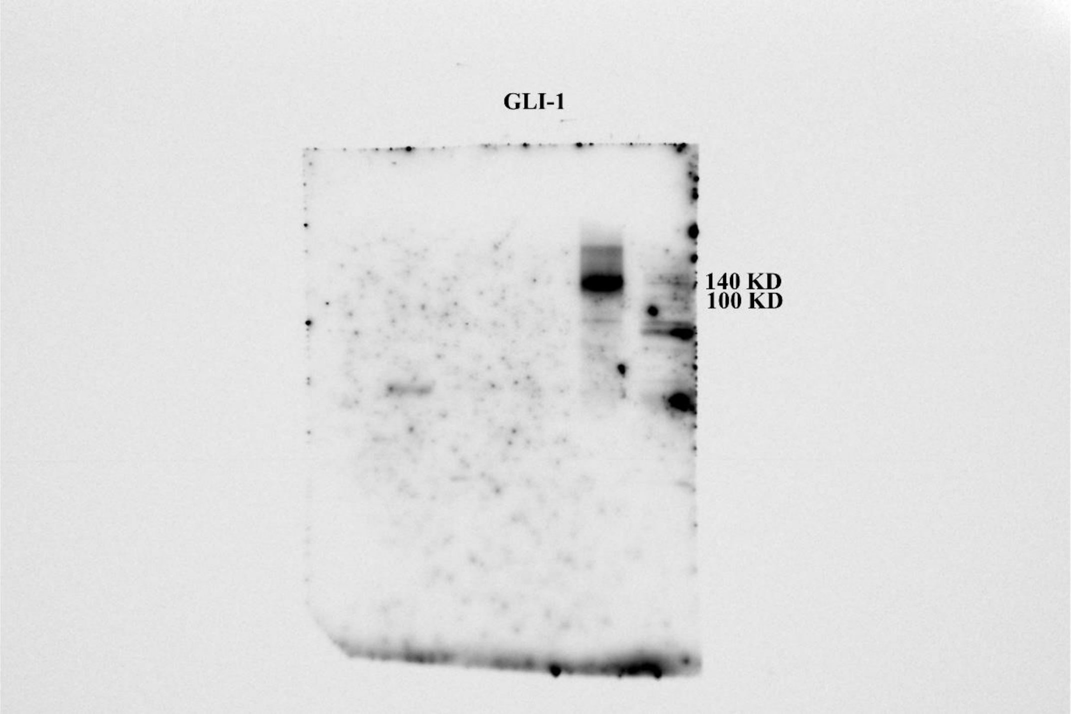

Supplemental Figure 2. Full-length blots of SHH(A) and GLI-1(B) expression in mammary gland tissue samples to check the size of each protein

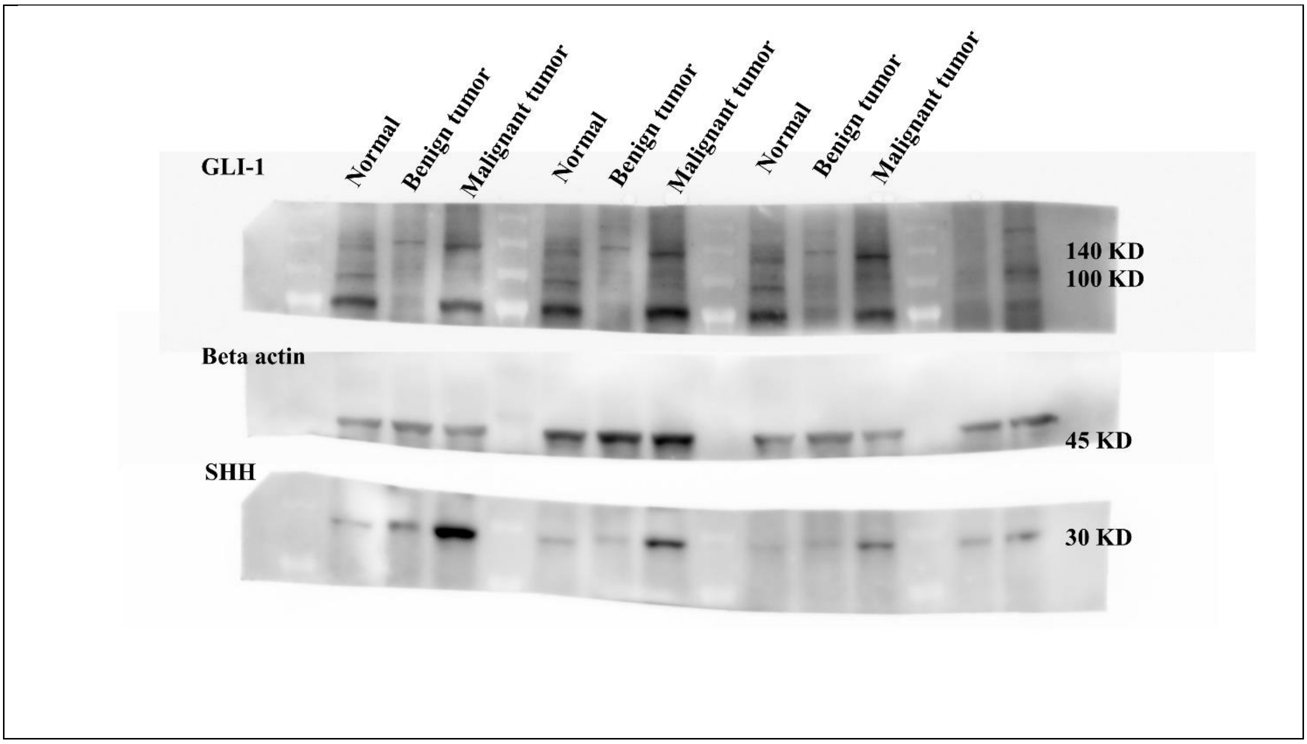

Supplemental Figure 2. Full-length blots of SHH(A) and GLI-1(B) expression in mammary gland tissue samples to check the size of each protein
