# Supplementary material for: Usability of serum hedgehog signalling proteins as biomarkers in canine mammary carcinomas
Source: BMC Vet Res. 2023 Nov 6;19:231. doi: 10.1186/s12917-023-03761-7 (PMC10626804; doi:10.1186/s12917-023-03761-7)
Supplement: Supplementary file 2 — Additional file 2. [file 12917_2023_3761_MOESM2_ESM.pdf]

The ARRIVE Essential 10: author checklist

The ARRIVE Essential 10

These items are the basic minimum to include in a manuscript. Without this information, readers and reviewers cannot assess the reliability of the findings.

Item		Recommendation	Section/line number, or reason for not reporting
**Study design**	1	For each experiment, provide brief details of study design including:	
		a. The groups being compared, including control groups. If no control group has been used, the rationale should be stated.	Materials and methods - Patient selection
		b. The experimental unit (e.g. a single animal, litter, or cage of animals).	Materials and methods - Patient selection
**Sample size**	2	a. Specify the exact number of experimental units allocated to each group, and the total number in each experiment. Also indicate the total number of animals used.	Materials and methods - Patient selection
		b. Explain how the sample size was decided. Provide details of any *a priori* sample size calculation, if done.	The appropriate number of samples were estimated with G*Power 3.1 software program.
**Inclusion and exclusion criteria**	3	a. Describe any criteria used for including and excluding animals (or experimental units) during the experiment, and data points during the analysis. Specify if these criteria were established *a priori*. If no criteria were set, state this explicitly.<br>b. For each experimental group, report any animals, experimental units or data points not included in the analysis and explain why. If there were no exclusions, state so.<br>c. For each analysis, report the exact value of *n* in each experimental group.	The animals that enrolled in this experiment were dogs with owners.
**Randomisation**	4	a. State whether randomisation was used to allocate experimental units to control and treatment groups. If done, provide the method used to generate the randomisation sequence.<br>b. Describe the strategy used to minimise potential confounders such as the order of treatments and measurements, or animal/cage location. If confounders were not controlled, state this explicitly.	The animals that enrolled in this experiment were dogs with owners.
**Blinding**	5	Describe who was aware of the group allocation at the different stages of the experiment (during the allocation, the conduct of the experiment, the outcome assessment, and the data analysis).	The first author was aware of group allocation.
**Outcome measures**	6	a. Clearly define all outcome measures assessed (e.g. cell death, molecular markers, or behavioural changes).<br>b. For hypothesis-testing studies, specify the primary outcome measure, i.e. the outcome measure that was used to determine the sample size.	The primary outcome: Western blot was done to confirm the expression of Hh signaling protein. Secondary outcomes: Serum levels of SHH and GLI-1 were measured using ELISA kits.
**Statistical methods**	7	a. Provide details of the statistical methods used for each analysis, including software used.	Materials and methods - Statistical analysis
		b. Describe any methods used to assess whether the data met the assumptions of the statistical approach, and what was done if the assumptions were not met.	Materials and methods - Statistical analysis
**Experimental animals**	8	a. Provide species-appropriate details of the animals used, including species, strain and substrain, sex, age or developmental stage, and, if relevant, weight.	Materials and methods - Patient selection
		b. Provide further relevant information on the provenance of animals, health/immune status, genetic modification status, genotype, and any previous procedures.	Materials and methods - Patient selection
**Experimental procedures**	9	For each experimental group, including controls, describe the procedures in enough detail to allow others to replicate them, including:	Materials and methods
		a. What was done, how it was done and what was used.	Materials and methods
		b. When and how often.	Materials and methods
		c. Where (including detail of any acclimatisation periods).	Materials and methods
		d. Why (provide rationale for procedures).	
**Results**	10	For each experiment conducted, including independent replications, report:	
		a. Summary/descriptive statistics for each experimental group, with a measure of variability where applicable (e.g. mean and SD, or median and range).	Results - Serum levels of SHH and GLI-1
		b. If applicable, the effect size with a confidence interval.	Results - Serum levels of SHH and GLI-1
